# 4,5-Dimethoxycanthin-6-one is a novel LSD1 inhibitor that inhibits proliferation of glioblastoma cells and induces apoptosis and pyroptosis

**DOI:** 10.1186/s12935-021-02434-5

**Published:** 2022-01-18

**Authors:** Wei Li, Bai-sheng Huang, Yuan-yuan Xiong, Li-jian Yang, Li-xiang Wu

**Affiliations:** 1 Department of Physiology, School of Basic Medical Sciences, Central South University, 110 Xiangya Road, Changsha City, Hunan Province China; 2 Department of Neurosurgery, The Second Affiliated Hospital of Nanchang University, Nanchang, China; 3 Department of Neurosurgery, The First Hospital of Changsha, Changsha, China

**Keywords:** 4, 5-Dimethoxycanthin-6-one, LSD1, Pyroptosis, Apoptosis, Glioblastoma

## Abstract

**Background:**

Glioblastoma is one of the most common fatal intracranial malignancies. Lysine-specific demethylase 1 (LSD1) reportedly has therapeutic effects on a variety of tumors. This study explored the therapeutic effect of LSD1 inhibition on glioblastoma cell lines and the possible underlying mechanisms.

**Methods:**

The MTT assay was utilized to screen for the sensitivity of U87, U251 and T98G cells to 4, 5-dimethoxycarrageenin-6-one. qRT-PCR and western blot were used to measure the proliferation, apoptosis, and pyroptosis signaling pathway expression to observe the effect of LSD1 inhibition on U251 and T98G cells. Flow cytometry, immunofluorescence, immunohistochemistry, wound scratch, clone formation, and TUNEL assay were used to analyze the effects of 4, 5-dimethoxycanthin-6-one on glioblastoma cells. The effect of 4, 5-dimethoxycanthin-6-one was examined in vivo in BALB/c nude mice injected with U251 cells. HE staining was used to detect the histopathology of the tumor.

**Results:**

LSD1 specifically catalyzes the demethylation of monomethylated and demethylated histone H3 lysine at position 4 (h3k4me1, h3k4me2, h3k4me3) and lysine at position 9 (h3k9me1). This regulated the transcriptional activity of proliferation, apoptosis, and pyroptosis signaling pathway genes. In vitro, the proliferation of glioblastoma cells was decreased in the 4, 5-dimethoxycanthin-6-one group. The expression of Caspase1 in glioblastoma cells treated with 4, 5-dimethoxycanthin-6-one increased, and the number of apoptotic cells increased. The tumor volume of mice injected with 4, 5-dimethoxycanthin-6-one decreased significantly.

**Conclusion:**

4, 5-Dimethoxycanthin-6-one could act as a novel inhibitor of LSD1 to regulate glioblastoma, which could inhibit the proliferation of U251 and T98G cells and induce their apoptosis and pyroptosis. It is a potential drug for the treatment of glioblastoma.

**Supplementary Information:**

The online version contains supplementary material available at 10.1186/s12935-021-02434-5.

## Introduction

Glioblastoma is one of the most aggressive cancers of the central nervous system. Glioblastoma has a poor prognosis [1] with a median survival time of 5–15 months [2]. The treatment failure and poor prognosis observed in the vast majority of patients with glioblastoma may be due to the high inherent resistance to chemotherapy and radiation, and the extremely rapid spread of glioblastoma cells throughout the brain [3]. As the most commonly used treatment for glioblastoma, surgical resection followed by concurrent chemoradiotherapy (CCRT) can prolong progression-free survival [4, 5]. However, CCRT t can result in serious adverse reactions that reduce the quality of life of patients. Specifically, frequent change targeting in glioblastoma cells, such as mammalian target protein phosphoinositide 3-kinase/protein kinase B/rapamycin (PI3K/AKT/mTOR), p53 and retinoblastoma (RB) pathways, epidermal growth factor receptor genes, and amplification or mutation failure can improve the prognosis. This may be due to the redundancy of the compensation mechanism and the insufficient coverage of some targets related to the blood–brain barrier or poor tolerance and safety [6]. Transcriptional activation and repression of tumorigenesis-relevant genes are influenced by local histone methylation. For example, methylation of histone 3 lysine 4 (H3K4) residues is associated with increased gene expression and is reduced in severe glioblastoma cases [7]. The study found that H4K20me3 and H3K4me3 RNAs co-localize in promoter and gene body regions of active genes with histone modifications such as H3K36me3 and transcriptional machinery. Moreover, H4K20me3 and H3K4me3 are associated with distinct gene features including transcripts of greater length and exon number relative to unoccupied transcripts [8]. H3K4me3 and H3K9me3 are unstable in methionine-addicted cancer cells, but stable in normal cells [9]. High expression of H4K20me3 was associated with good prognosis in colon cancer [10]. Therefore, the catalytic enzymes of these non-permanent epigenetic markers are of particular interest in therapeutic approaches.

Lysine-specific demethylase 1 (LSD1) is overexpressed in many human cancers and is often associated with more aggressive tumors with poor prognosis [11, 12]. LSD1 knockdown significantly promotes cancer cell apoptosis, inhibits anti-apoptotic BCL-2 protein expression, and increases the expression of the pro-apoptotic protein BAX [13]. Comprehensive examinations of LSD1 have gradually revealed the details of the downstream signaling pathway regulated by LSD1. Loss of LSD1 demethylase activity can cause senescence of trophoblast stem cells [14] and prevent age-programmed loss of beige fat cells [15]. LSD1 acts as a co-activator or co-inhibitor by methylating lysine 4 on histone H3 (H3K4me/me2) and H3 lysine 9 dimethylation (H3K9me/me2) [16]. Some reports proved that LSD1 could regulate the PI3K/Akt/mTOR pathway by combining the promoter region of tumor suppressor PTEN and inducing autophagy in ovarian cancer cells [17, 18]. A study showed that inhibition of LSD1 decreased the expressions of related protein, not only in the Notch pathway (such as Notch3, Notch1, DTX1 and Hes1), but also in the PI3K/Akt/mTOR pathway such as PI3K, p-Akt (Ser473), p-mTOR (Ser2448), p-mTOR (Ser2481) and Rictor in ESCC cells, which indicated that LSD1 might have positively regulating effects on the Notch and PI3K/Akt/mTOR signaling pathways in ESCC [19]. The mTOR pathway is dysregulated in several human diseases, including cancer and diabetes, as well as intellectual disabilities, such as FXS, Rett syndrome and ASD. Thus, there is a wealth of evidence implicating mTOR and MAPK pathways, and ultimately protein synthesis, in syndromic ASD [20]. However, it is unclear whether LSD1 can regulate the AKT/mTOR and mitogen-activated protein kinase (MAPK) signaling pathways to affect the proliferation of glioblastoma cells. The pathway of glioblastoma cell apoptosis is also unclear. Pyroptosis is a pro-inflammatory and lytic mode of cell death. This cell death is dependent on Caspase1 activation and leads to a series of reactions that ultimately affect cells [21, 22]. Whether inhibition of LSD1 expression leads to apoptosis of glioblastoma cells requires further verification.

Many natural products from plants have shown good potential for the treatment or prevention of cancer. The active substances in plants are less toxic to the human body. 4,5-dimethoxycanthin-6-one is one of the main active canthaxanthin alkaloids isolated from quassinoids of *Picrasma quassiodes*. 4,5-dimethoxycanthin-6-one has anti-inflammatory, antiviral, antihypertensive, and anticancer activities [23–25]. 4,5-dimethoxycanthin-6-one may impede the progression of glioblastoma by LSD1-mediated epigenetic (heritable non-DNA sequence changes) modifications that affect key transcription factors. These changes in turn control the transition between carcinogenic states [26]. Substances have been identified that affect the migration, invasion, proliferation, and apoptosis of glioblastoma cells by regulating LSD1 [27, 28]. Our previous data confirmed that 4,5-dimethoxycanthin-6-one could inhibit the expression of LSD1. Whether 4,5-dimethoxycanthin-6-one affects the proliferation, apoptosis, and pyroptosis of glioblastoma cells is unclear and warrants investigation.

In the present study, we hypothesized that the inhibition of LSD1 by 4, 5-dimethoxycanthin-6-one leads to the inhibition of the AKT/mTOR and MAPK proliferation pathways of U251 and T98G cells, upregulation of the expression of the BAX pro-apoptotic gene, and activation of Caspase1 to promote the apoptosis of U251 and T98G cells. The results of this study demonstrated that 4, 5-dimethoxycanthin-6-one can inhibit the development of U251 and T98G cells by regulating LSD1, implicating LDS1 as a valuable target in the treatment of glioblastoma. We added a hypothesis figure for better understanding (Additional file 1: Fig. S1).

## Material and methods

### Cell culture and treatment

The HKF, U87, U251 and T98G glioblastoma cell lines and lentivirus were purchased from HonorGene (Changsha Aibiwei Biotechnology Co., Ltd.). The cells were cultured in Dulbecco’s modified Eagle’s medium (DMEM) containing 10% fetal bovine serum. Cell culture conditions were 37 °C and 5% CO_2_. U87, U251, and T98G cells were treated with a concentration gradient of 0–4 μM 4, 5-dimethoxycanthin-6-one to screen out sensitive cell. Cell lines were grouped into the Control group (U251 or T98G cells), 1 μM group (U251 or T98G cells with 1 μM 4,5-dimethoxycanthin-6-one), 2 μM group (U251 or T98G cells with 2 μM 4,5-dimethoxycanthin-6-one), 4 μM group (U251 or T98G cells with 4 μM 4,5-dimethoxycanthin-6-one), short hairpin (sh)-NC (T98G cells transfected with sh-NC lentivirus), and sh-LSD1 group (T98G cells transfected with sh-LSD1 lentivirus). T98G cells in sh-NC and sh-LSD1 groups were treated with 0–16 μM 4, 5-dimethoxycanthin-6-one. The LSD1 knock-down in T98G cells was carried out by transfecting 40 pmol SMART pool LSD1 siRNAs (Cat#:M-009223-00-0050, Dharmacon RNAi technologies) using Nucleofector II. The cells were analyzed in 48 h post-transfection. The siRNA sequences are available in the Additional file 5: Table S1. The adherent cells were placed in a 24 well plate at 1 × 10^5^/well. The number of cells transfected by lentivirus reached about 2 × 10^5^/well. On the second day, we replaced the original medium with 2 mL fresh medium containing 6 μg/ml polybrene, and then added an appropriate amount of virus suspension and continued culture for 24 h. When the cells are stable and passed on to the third generation. Cells were collected for subsequent tests.

### Viability assay

The U87, U251 and T98G were digested with trypsin digestion solution, and a cell suspension was prepared. The suspended cells were inoculated into wells of 96-well plates (1 × 10^4^ cells/100 μL), with three wells used in each group. After 24 h incubation with above indicated compounds, 10 μL/well MTT (5 mg/ mL, HG-M100, Honorgene, China) to each well and incubated at 37 ℃ with 5% CO2 for 4 h. The supernatant was centrifuged, and then 150 μL/well dimethyl sulfoxide was added. The Optical Density (OD) value at 490 nm was analyzed by a Bio-Tek enzyme plate analyzer (MB-530, Heaples, China). Then we treated HKF, U251 and T98G cells with the same method. After the compounds were incubated for 12, 24 and 48 h, 10 μL/well MTT was added to each well and incubated at 37 ℃ with 5% CO_2_ for 4 h. The Optical Density (OD) value at 490 nm was analyzed by a Bio-Tek enzyme plate analyzer.

### Quantitative real-time PCR (qRT-PCR)

About 0.02 g of tissue stored in Trizol was put into a new 1.5 mL centrifuge tube. 1 mL Trizol was added to the homogenizer (BioPrep-24, Allsheng China) to grind the homogenate thoroughly. After mixing, the homogenate was cracked in the chamber for 3 min. Total RNA of glioblastoma was extracted by Trizol (15596026, Thermo, USA). The sample RNA was reverse transcribed to cDNA using the cw2569 kit according to the instructions of the manufacturer (Kangwei Century Company, China). The reaction conditions comprised 40 cycles of denaturation at 95 °C for 10 min followed by 94 °C for 15 s and annealing at 60 °C for 30 s. The internal reference primer was β-actin. The primer sequences are presented in Table 1. With 2 μg cDNA as template, the 2^−△△Ct^ relative quantitative method. The relative transcription level of the target gene was calculated as: △△Ct = △ experimental group − △ Control group, △Ct = Ct (target gene) − Ct (β-actin).

**Table 1:** Primer sequences

Gene	Forward (F) and Reverse (R) Sequences (5ʹ–3ʹ)
BAX	F: TGAAGACAGGGGCCTTTTTG
	R: AATTCGCCGGAGACACTCG
IL-1β	F: CAGAAGTACCTGAGCTCGCC
	R: AGATTCGTAGCTGGATGCCG
BCL-2	F: GGACCGCGTATCAGAGCTTT
	R: CAGTGCCCCGCCAAAGGA
Caspase-3	F: TCTGACTGGAAAGCCGAAACTCT
	R: AGCCATCTCCTCATCAGTCCCA
XIAP	F: TGCTGGACTGGGCTCCCTAT
	R: CACGAAGTTTAAGGAGGCTGACC
NLRP3	F: CCTCTTTGGCCTTGTAAACCAG
	R: TGGCTTTCACTTCAATCCACT
Caspase-1	F: ACAAGGCACGGGACCTATG
	R: TCCCAGTCAGTCCTGGAAATG
β-actin	F: ACATCCGTAAAGACCTCTATGCC
	R: TACTCCTGCTTGCTGATCCAC

### Western blot

The model R0010 total protein extract RIPA kit (Solarbio, China) was used to extract total protein from glioblastoma cells. The bicinchoninic acid method was used to determine the protein concentration. The membrane was blocked with 5% skimmed milk for 2 h to allow non-specific binding prior to incubation with the primary antibody overnight at 4 °C. Rabbit anti-H3K4me1 (1:600, ab8895), rabbit anti-H3K4me2 (1:500, ab32356), mouse anti-H3K9me2 (1:5000, ab1220), rabbit anti-H3K4me3 (1:1000, ab8580), rabbit anti-p-c-Raf (1:2000, ab173539), and rabbit anti-p-MEK1 (1:2000, ab96379) were purchased from Abcam (UK). Rabbit anti-LSD1 (1:2000, 20813-1-AP), rabbit anti-H3 (1:3000, 17168-1-AP), rabbit anti-mTOR (1:500, 66888-1-Ig), rabbit anti-p-mTOR (1:500, 67778-1-Ig), rabbit anti-AKT (1:1000, 10176-2-AP), mouse anti-p-AkT (1:5000, 66444-1-Ig), mouse anti-c-Raf (1:300, 66592-1-Ig) and rabbit anti-MEK1 (1:2000, 51080-1-AP), rabbit anti-BAX (1:6000, 50599-2-Ig), rabbit anti-Cleaved-caspase3 (1:2000, 19677-1-AP), rabbit anti-BCL-2 (1:2000, 12789-1-AP), rabbit anti-XIAP (1:1000, 10037-1-Ig), rabbit anti-NLRP3 (1:600, 19771-1-AP), rabbit anti-Caspase1 (1:1000, 22915-1-AP), rabbit anti-IL-1β (1:1000, 16806-1-AP), rabbit anti-IL-18 (1:2000, 16663-1-AP), and mouse anti-β-actin (1:5000, 66009-1-Ig) were purchased from the Proteintech (USA). The membrane was immersed in Superecl Plus (k-12045-d50, Advansta, USA) for the development of luminescence. β-Actin was used as an internal reference. Image J software (NIH, USA) was used to analyze gray values.

### LSD1 enzyme assay

The Epigenase™ LSD1 Demethylase Activity/Inhibition Assay Kit (Colorimetric) contains all reagents necessary for the measurement of LSD1 activity/inhibition. In this assay, di-methylated histone H3-K4 LSD1 substrate is stably coated onto the strip wells. Active LSD1 binds to the substrate and removes methyl groups from the substrate. The LSD1-demethylated products can be recognized with a specific antibody. The ratio or amount of demethylated products, which is proportional to enzyme activity, can then be colorimetrically measured by reading the absorbance in a colorimetric microplate reader at a wavelength of 450 nm. The activity of LSD1 enzyme is proportional to the optical density intensity measured.

### Wound scratch assay

The cells transfected for 48 h in each group were inoculated into 6-well plates. Each group was examined using triplicate wells. Cells were grown to 90% confluence, washed once with sterile PBS, scratched with a sterile pipette tip, and washed once with PBS. The dislodged cells were removed, and serum-free DMEM high glucose medium (d5796-500 ml, Sigma-Aldrich, China) was added for continuous culture. Images were obtained at various times using a model DSZ2000x inverted biological microscope (Cnmicro, Beijing).

### Flow cytometry

Cells were collected by centrifugation at 1500×*g* for 5 min and washed once with PBS (SH30256.01, Hyclone, USA). Five microliters of Annexin V-FITC (KGA108, Keygen, China) was added to the mixture. Propidium iodide (5 μL) was then added and mixed. All steps were performed in a dark room.

### Colony formation assay

The cells were trypsinized to prepare a cell suspension. The suspended cells were seeded in a 6-well plate at 2 × 10^5^ cells/well. After overnight culture the cells were washed twice with PBS for drug intervention. After intervention for 24 h, the cultured cells were digested with 0.25% trypsin digestion solution (C0201, Beyotime, China) and suspended in a serum-free basal medium. The cell density was adjusted to approximately 1 × 10^5^/mL. In each group, 2 mL containing 1000 cells was inoculated in each well of a 6-well plate. The plates were gently agitated to disperse the cells. The cells were cultured in a 37 °C, 5% CO_2_ incubator (DYY-6C, LIUYI, China) with replacement of the medium was changed every 2–3 days. The culture medium was discarded and the cells were washed twice with PBS. The washed cells were fixed with 4% paraformaldehyde (P0099, Beyotime, China) for 30 min, washed twice with PBS, stained for 5 min with 0.5% crystal violet (C0121, Beyotime, China), and washed with aseptic water more than three times. Each well was photographed and the number of clones in each well was counted.

### Cell proliferation assay

Following the particular treatment, ethynyldeoxyuridine (EDU) solution was diluted with cell culture medium at a ratio of 1000:1 to prepare the 50 μM EDU working solution. 20 mg/mL glycine (AWT0119a, Wellbio, China) was added to each well. The EDU working fluid was preheated at 37 °C and equal volumes were added to wells of a 96-well plate. The plate was incubated at 37 °C and 5% CO_2_ for 2 h. The cells were fixed with 4% paraformaldehyde for 15 min and permeabilized with 0.3% Triton X-100 for another 15 min at room temperature. Each well was incubated with 60 μL of the click additive solution for 30 min at room temperature in the dark. One hundred microliters of 1 × Hoechst 33342 reaction solution was added to each well. An average EDU intensity greater than 500 was considered EDU-positive. The ratio of EDU-positive cells to total cells was calculated as the percentage of EDU-positive cells.

### Immunofluorescence (IF) assay

Cultured cells (T98G or U251) were fixed for 15 min in 4% paraformaldehyde in PBS (AWC0216a, Wellbio, China) and 10 min in methanol. Cells were washed with PBS three times for 3 min each time, and incubated with bovine serum albumin (BSA, 5%) at 37 °C for 60 min. Primary antibody was added and incubated at 4 °C overnight. The primary antibodies targeted Caspase 1, glial fibrillary acidic protein (GFAP; 1:50, 22915-1-AP, Proteintech) + interleukin (IL)-18 (1:50, 10663-1-AP, Proteintech), and GFAP + IL-1 β (1:50, ab4648, Abcam) were dripped. PBS was washed three times for 5 min and then incubated at 37 °C for 90 min with the corresponding secondary antibodies. The antibodies were CoraLite594-conjugated goat anti-mouse IgG (H + L) (1:200, SA00013-3, Proteintech) and CoraLite594-conjugated goat anti-rabbit IgG (H + L) (1:200; SA00013-2, Proteintech). A solution of 4ʹ,6-diamidino-2-phenylindole (DAPI) (AWC0291a, Wellbio, China) was used to stain nuclei at 37 °C for 10 min, followed by washing with PBS was used to wash the nucleus 3 times, 5 min each. The cells were observed in the dark using a model BA210T fluorescence microscope (Motic, China).

### Terminal deoxynucleotidyl transferase dUTP nick end labeling (TUNEL) assay

Sections were deparaffinized in water, placed in xylene solution for 20 min each time, followed by sequential exposure to 100%, 95%, 85%, and 75% ethanol for 5 min at each concentration. The sections were then soaked in distilled water for 5 min. Each sample was prepared in a ratio of 99 μL of 1 × PBS to 1 μL of 100 × proteinase K. Each sample received drops of 100 μL proteinase K working solution and reacted at 37 °C for 20 min. After rinsing with PBS three times for 5 min each time, cell nuclei were stained with DAPI at 37 °C for 10 min. Each section was rinsed three times with PBS for 5 min each time, sealed with buffered glycerin, and examined by fluorescence microscopy.

### Nude mouse xenograft experiment

Male BALB/c nude mice were purchased from Hunan SJA Laboratory Animal Co., Ltd. The mice (n = 16, 16–20 g) were injected with 1 × 10^7^ U251 cells into their dorsal flanks. Eight mice were not treated further and comprised the Control group. The remaining 8 were used as the 4 μM group. The 4 μM group processing method was as follows: they were given 4 μM 4, 5-Dimethoxycanthin-6-one (50 mg/kg) orally after injection of 1 × 10^7^ U251 cells. From the 10th day after inoculation, the length and width of tumor xenografts were measured every 3 days. The tumor volume was calculated as width^2^ × length × 0.5. The mice were sacrificed on day 28 and the xenografts were weighed and dissected.

### Immunohistochemistry (IHC)

Primary antibody to LSD1 (ab129195, Abcam) was added and incubated overnight at 4 °C. PBS was rinsed three times for 5 min each time. Anti-Rabbit IgG-HRP (50–100 μL, Bio-Rad, USA) was added and incubated at 37 °C for 30 min, then washed with PBS three times for 5 min each. The samples were than exposed to two xylene solutions for 10 min each time. Each samples was sealed using neutral gum and examined by microscopy.

### Hematoxylin–eosin (HE) staining

The xenografts were washed with normal saline and fixed in 4% paraformaldehyde for 15 min, and then were washed, dehydrated, permeabilized, waxed, embedded, and sliced. After that, the tissue sections were put on the slide, dried in a 45 °C incubator, dewaxed, soaked in gradient ethanol, and washed with distilled water for 5 min. Stained with hematoxylin (AWI0009a, Wellbio, China) for 3 min, the sections were washed with running water for 3 s, differentiated with 1% hydrochloric acid ethanol for 3 s, and stained with 5% eosin solution for 3 min. Subsequently, the sections were dehydrated, permeabilized, sealed, and observed under a microscope (Motic, China).

### Statistical analyses

All data were analyzed using GraphPad Prism 8.0 software (GraphPad Software, USA). Unpaired t-test was used to compare the two groups conforming to the normal distribution. Comparisons among multiple groups were conducted using one-way analysis of variance (ANOVA), followed by Tukey’s post hoc test. Differences were considered statistically significant at P < 0.05.

## Results

### 4, 5-Dimethoxycanthin-6-one is a novel LSD1 inhibitor

**Fig. 1:**
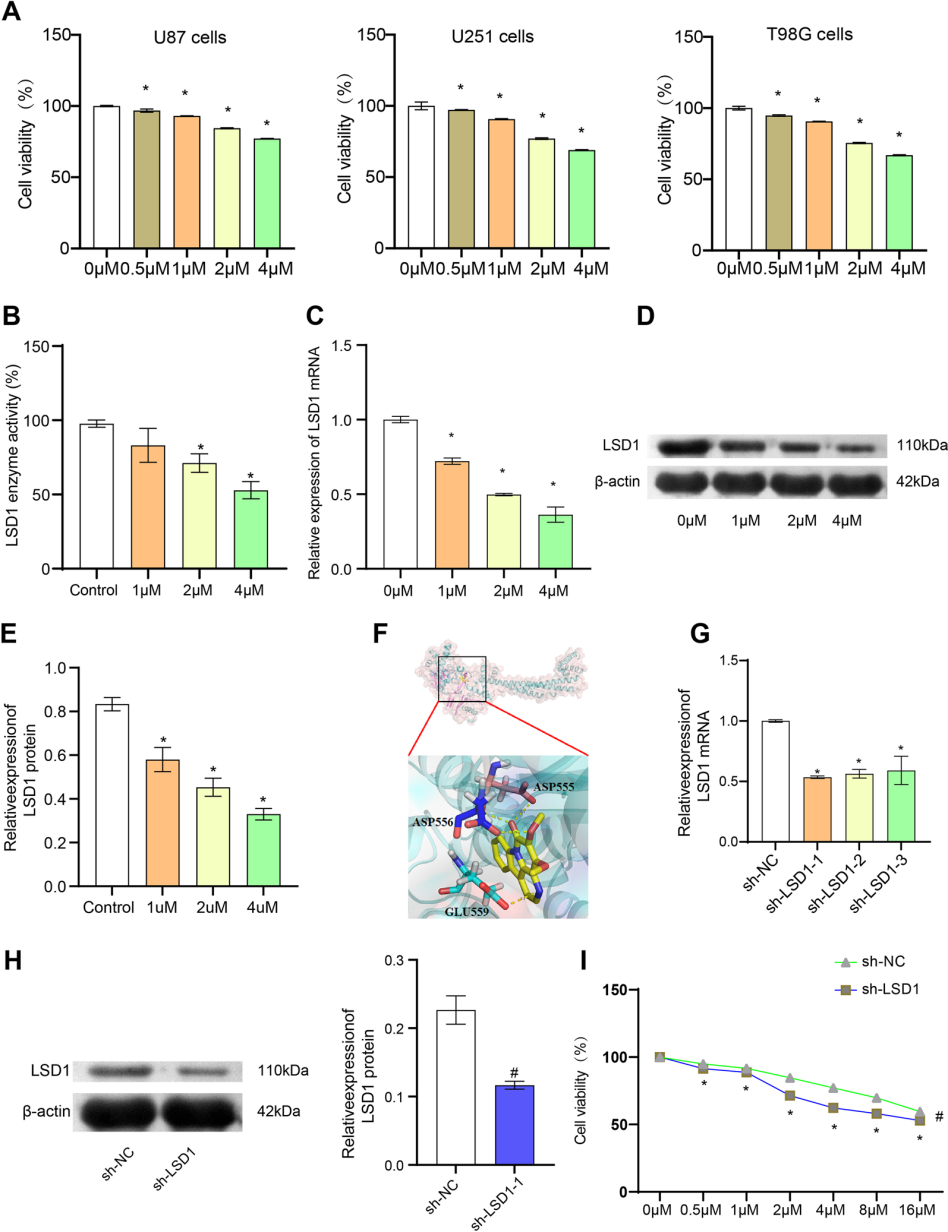
4, 5-Dimethoxycanthin-6-one inhibits LSD1. **A** Viability of T98G, U87, and U251 cells. **B** Dose-dependent change in LSD1 enzyme activity. **C**–**E**. LSD1 levels in the 0, 1, 2, and 4 μM groups. **F** Three-dimensional binding model of the LSD1 active site 4 of 4,5-dimethoxycanthin-6-one (PDB code: 5l3e). **G**, **H** Relative LSD1 expression of sh-LSD1 and sh-NC cells. **I** Anti-proliferation effect of 4,5-dimethoxycanthin-6-one on sh-LSD1 and sh-NC cells. *P < 0.05 compared with the 0 μM group, #P < 0.05 compared with the sh-NC group

Recent studies reported that LSD1 is aberrantly overexpressed in glioblastoma and is associated with tumorigenesis [7, 29]. Based on the most effective inhibition of LSD1 activity, 4, 5-dimethoxycanthin-6-one was evaluated for its anticancer activity in vitro against T98G, U87, and U251 glioblastoma cells. The cells were treated with 4, 5-dimethoxycanthin-6-one for 24 h and viability was assessed using the MTT assay. As shown in Fig. 1A, 4, 5-dimethoxycanthin-6-one exhibited potent anticancer activity in vitro against T98G and U251 cells. We have added MTT assay to detect the toxicity of 4, 5-dimethoxycanthin-6-One to HKF cell lines. 4, 5-dimethoxycanthin-6-One had a slight inhibitory effect on HKF cell lines activity (Additional file 2: Fig. S2). The viability results combined with the LSD1 enzyme assay, qRT-PCR and western blot results indicated that 4,5-dimethoxycanthin-6-one effectively inhibited LSD1 activity in a concentration-dependent manner (Fig. 1B–E). As shown in Fig. 1F, 4, 5-dimethoxycanthin-6-one (colored yellow) occupied the cavity of LSD1. The carbonyl group of 4, 5-dimethoxycanthin-6-one formed two hydrogen bonds with ASP555. The nitrogen atom of the pyridine ring formed a hydrogen bond with the GLU559. The methoxy group of 4, 5-dimethoxycanthin-6-one formed a hydrogen bond with ASP556. Binding models indicated that 4, 5-dimethoxycanthin-6-one could inhibit LSD1 activity. The LSD1 gene was silenced of T98G cells (sh-LSD1) and Control cells (sh-NC) to study the anti-proliferative activity of 4,5-dimethoxycanthin-6-one in vitro (Fig. 1G, H). 4,5-dimethoxycanthin-6-one inhibited the proliferation of sh-NCs in a concentration-dependent manner. In contrast, 4,5-dimethoxycanthin-6-one showed worse inhibitory activity in the sh-LSD1 group (Fig. 1I). These results indicated that LSD1 might affect the antiproliferative effect of 4,5-dimethoxycanthin-6-one in glioblastoma cells.

### Enrichment effect of 4, 5-dimethoxycanthin-6-one on methylated H3 peptide substrate in glioblastoma cells

**Fig. 2:**
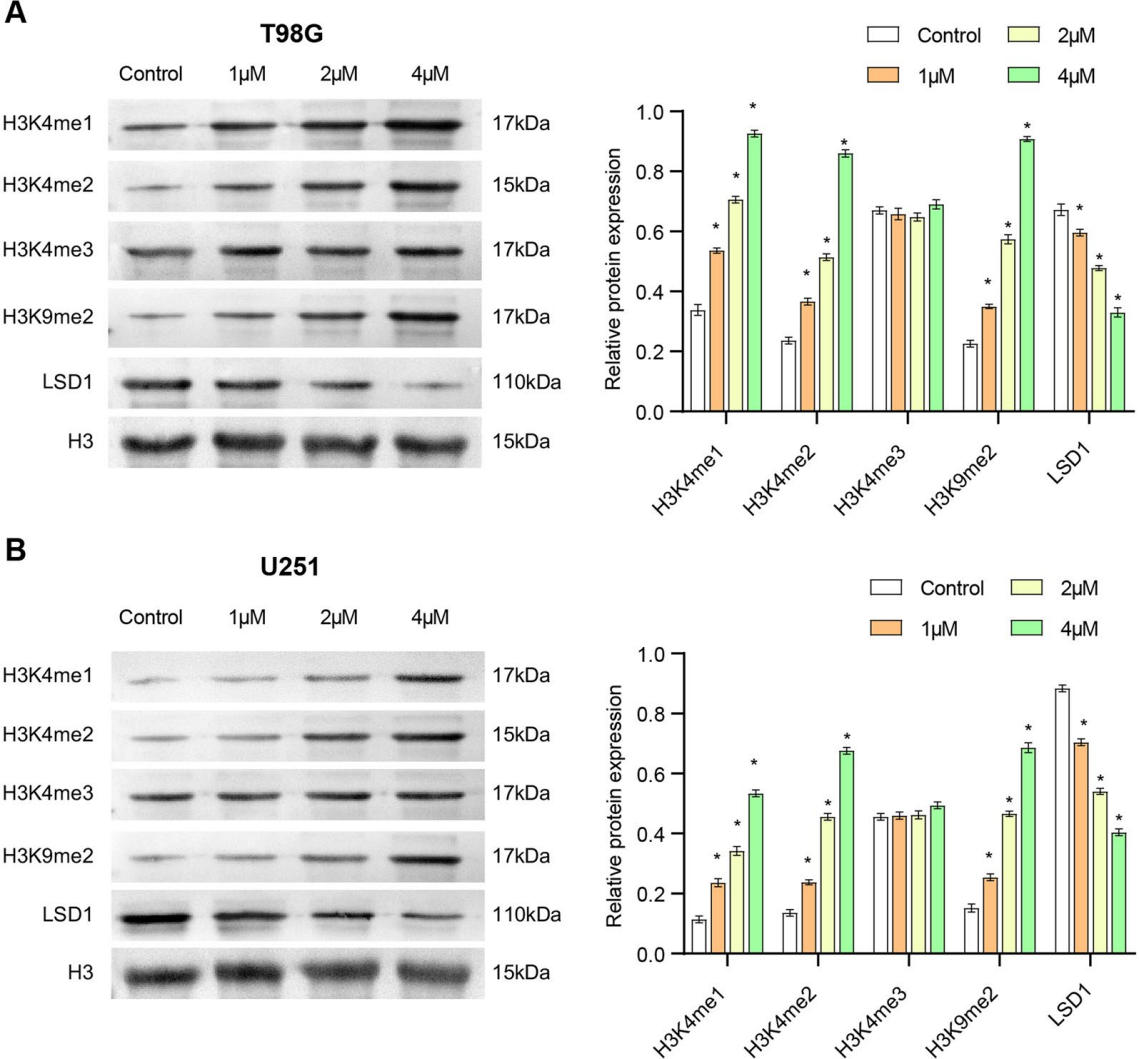
4, 5-Dimethoxycanthin-6-one targets glioblastoma cells. **A**, **B** Expression and histone methylation of LSD1 in T98G (**A**) and U251 (**B**) cells. *P < 0.05 compared with the Control group

Based on the anti-LSD1 activity of 4, 5-dimethoxycanthin-6-one, we studied the effect of its accumulation on methylated H3 peptide substrates in glioblastoma cells. We check the UCSC genome browser and see the chromatin enrichment marks on LSD promoter. The results showed that LSD1 is associated with H3K4 and H3K9, suggesting that there may be a targeting relationship (Additional file 3: Fig. S3). As shown in Fig. 2A, 4, 5-dimethoxycanthin-6-one induced enrichment of H3K4me1/2 and H3K9me2 and the degradation of LSD1 in T98G cells in a concentration-dependent manner. In addition, the expression levels of H3K4me3 remained unchanged. Figure 2B shows that the same situation was observed for the U251 and T98G cells. These results verified that 4, 5-dimethoxycanthin-6-one was the inhibitory target of LSD1 in glioblastoma cells. 4, 5-Dimethoxycanthin-6-one could inhibit LSD1 via methylation.

### 4, 5-Dimethoxycanthin-6-one is a novel LSD1 inhibitor that inhibits the AKT/mTOR and MAPKsignaling pathways in glioblastoma cells

**Fig. 3:**
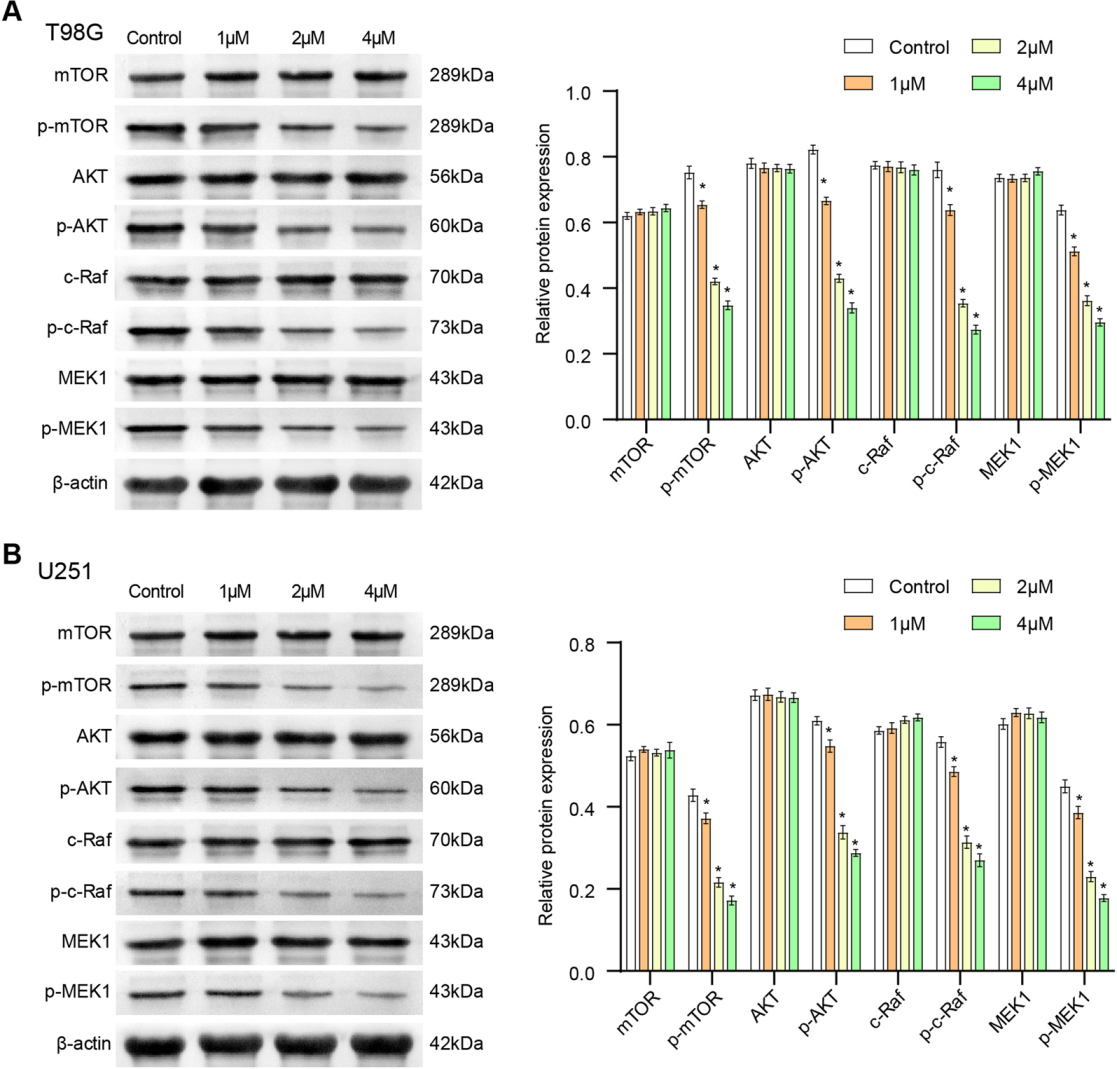
4, 5-Dimethoxycanthin-6-one inhibits the AKT/mTOR and MAPK signaling pathways. **A** 4, 5-Dimethoxycanthin-6-one inhibition of the AKT/mTOR and MAPK signaling pathways in U251 cells. **B** 4, 5-Dimethoxycanthin-6-one inhibition of the AKT/mTOR and MAPK signaling pathways in T98G cells. *P < 0.05 compared with the Control group

Next, we explored whether the proliferation of glioblastoma cells was affected. The AKT/mTOR and MAPK signaling pathways play a key role in cell proliferation. The results of western blot analysis are shown in Fig. 3A. In T98G cells, 4, 5-dimethoxycanthin-6-one had no obvious effect on the expression of mammalian target of rapamycin (mTOR), AKT, c-Raf, and MAPK kinase 1 (MEK1). Both p-AKT (473Ser) and p-mTOR (2448Ser) were downregulated by 4, 5-dimethoxycanthin-6-one. The expression levels of key proteins in the MAPK signaling pathway (p-c-Raf and p-MEK1) were also suppressed. Figure 3B displays the same situation in U251 and T98G cells. The above results showed that 4, 5-dimethoxycanthin-6-one can inhibit the AKT/mTOR and MAPK signaling pathways in a concentration-dependent manner.

### 4, 5-Dimethoxycanthin-6-one inhibits the proliferation of U251 and T98G cells

**Fig. 4:**
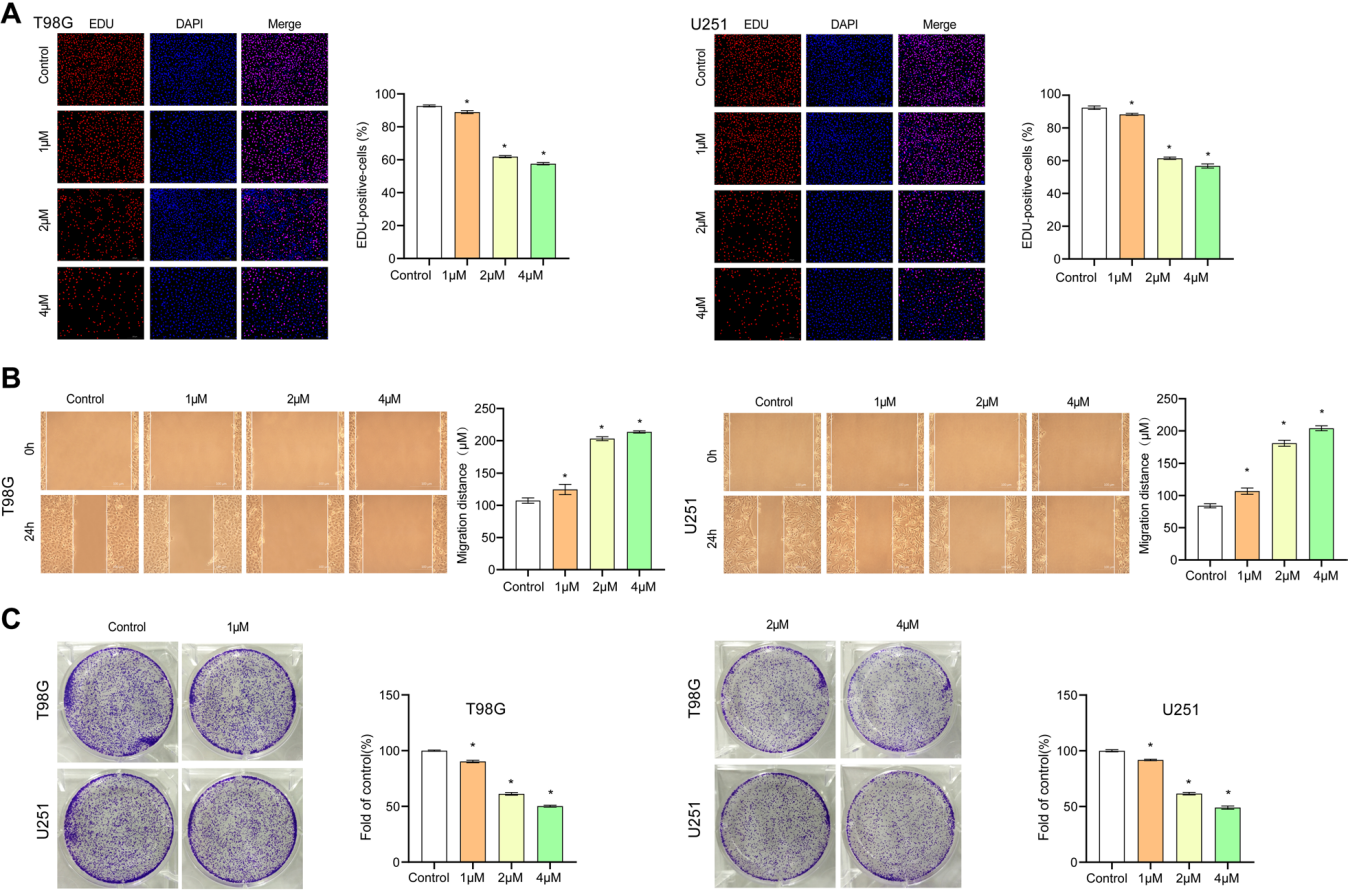
4, 5-Dimethoxycanthin-6-one inhibits cell proliferation. **A** Cell proliferation detected using the EDU assay. **B** The migration distance of cells was measured using a wound scratch assay. **C** Colon numbers were analyzed using a colony formation assay. *P < 0.05 compared with the Control group

Based on the above signaling pathway results, we speculated that 4,5-dimethoxycanthin-6-one can inhibit the proliferation of U251 and T98G cells. As shown in Fig. 4A, 4 μM 4,5-dimethoxycanthin-6-one inhibited the proliferation of U251 and T98G cells. Compared with the Control, the results of the scratch test indicated that migration of U251 and T98G cells was inhibited by 4 μM 4,5-dimethoxycanthin-6-one (Fig. 4B). The number of colonies was reduced (Fig. 4C). The collective data indicated that 4,5-dimethoxycanthin-6-one could inhibit the proliferation of U251 and T98G cells.

### 4, 5-Dimethoxycanthin-6-one promotes apoptosis of U251 and T98G cells

**Fig. 5:**
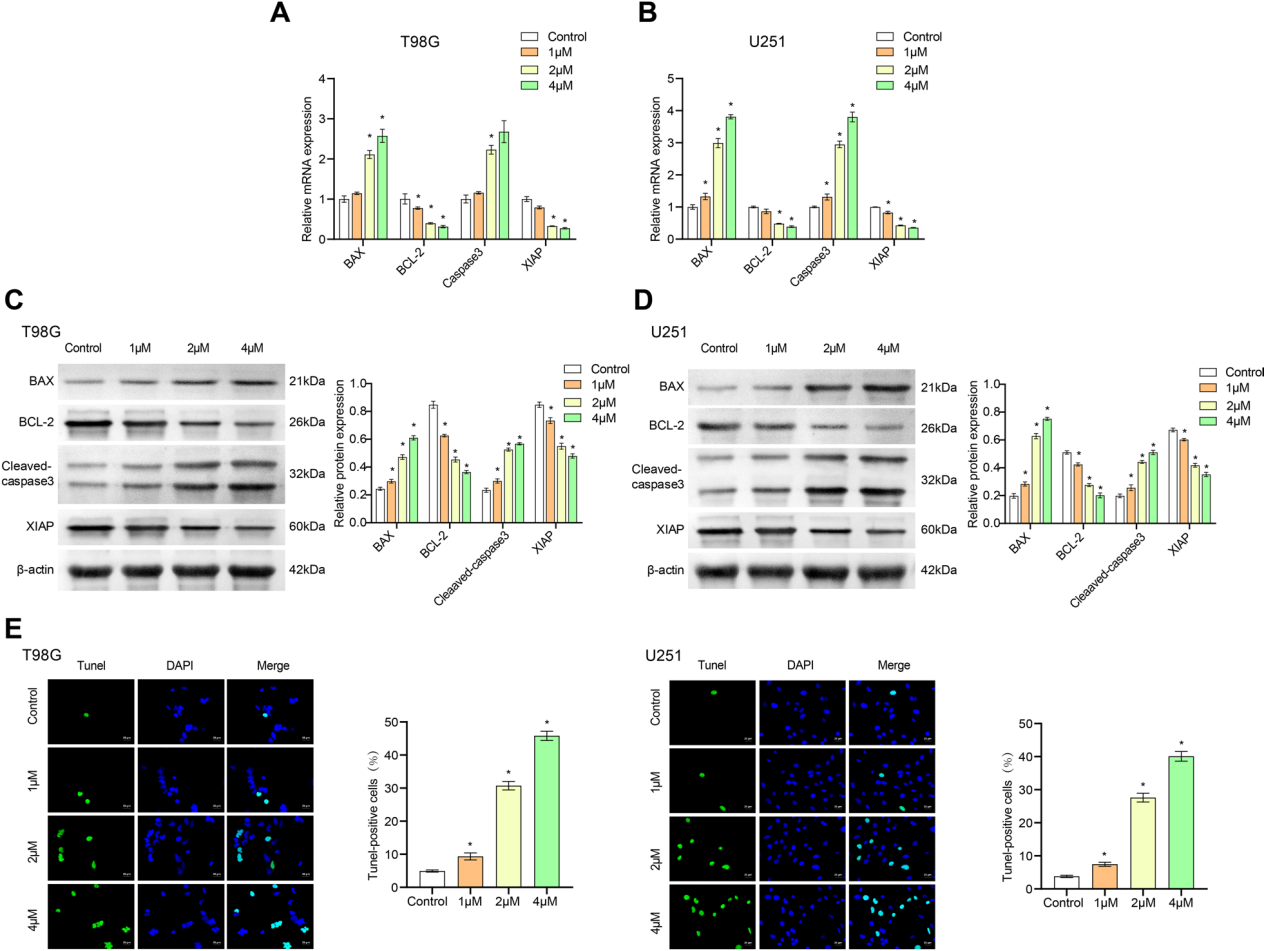
4, 5-Dimethoxycanthin-6-one promotes cell apoptosis. **A**–**D** Expression of apoptotic signaling pathway factors in qRT-PCR and western blot analyses. **E** Examination of the rate of apoptosis using the TUNEL assay. *P < 0.05 compared with the Control group

Next, we explored whether the apoptosis of glioblastoma cells was affected. The expression of pro-apoptotic genes BAX and Cleaved-caspase3 increased in the 4 μM 4,5-dimethoxycanthin-6-one group, while the expression of anti-apoptotic genes BCL-2 and XIAP decreased (Fig. 5A–D). The number of TUNEL-positive cells was increased in the 4 μM 4,5-dimethoxycanthin-6-one group. The results of the TUNEL assay (Fig. 5E) suggested that 4,5-dimethoxycanthin-6-one promoted the apoptosis of U251 and T98G cells in a concentration-dependent manner. Finally, the flow cytometry results in Additional file 4: Fig. S4 showed that 4,5-dimethoxycanthin-6-one might promote the rate of apoptosis of U251 and T98G cells.

### 4, 5-Dimethoxycanthin-6-one promotes the pyroptosis of U251 and T98G cells

**Fig. 6:**
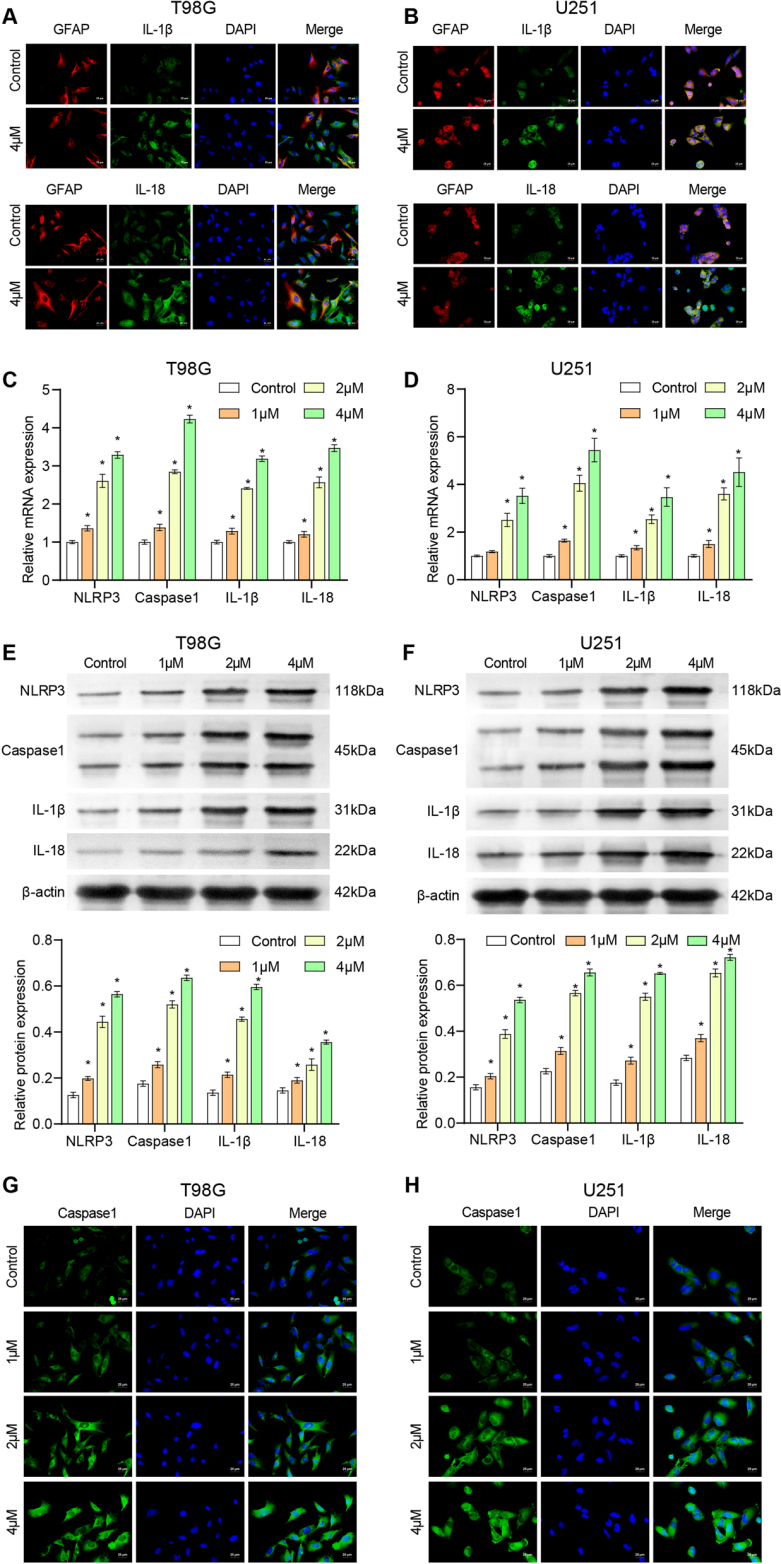
4, 5-Dimethoxycanthin-6-one is associated with cell pyroptosis. **A** The fluorescence intensities of GFAP, IL-1β, GFAP, and IL-18.**C**–**F**. qRT-PCR and western blot were used to identify the pyroptosis signaling pathway and the expression of inflammatory factors. **G** and **H**. IF detection of Caspase 1 in T98G (**G**) and U251 (**H**) cells.*P < 0.05 compared with the Control group

Pyroptosis is a highly inflammatory cell death pattern. IF was performed to observe the co-localization of the IL-1β and IL-18 pro-inflammatory cytokines with the GFAP glioblastoma marker. The IL-1β and IL-18 expression was increased in the 4 μM 4, 5-dimethoxycanthin-6-one group compared to that in the Control group (Fig. 6A, B). The qRT-PCR and western blot results revealed increased expression of NLR family pyrin domain containing 3 (NLRP3), Caspase1, IL-1β, and IL-18 in the 4 μM 4,5-dimethoxycanthin-6-one group. The findings suggested that 4, 5-dimethoxycanthin-6-one could activate the pyroptosis signaling pathway (Fig. 6C–F). The level of Caspase 1 was detected by IF. 4, 5-Dimethoxycanthin-6-one could promote the expression of Caspase1 (Fig. 6G, H). The collective findings indicated that 4, 5-dimethoxycanthin-6-one induced pyroptosis in U251 and T98G cells.

### 4, 5-Dimethoxycanthin-6-one inhibits development of glioblastoma in nude mice

**Fig. 7:**
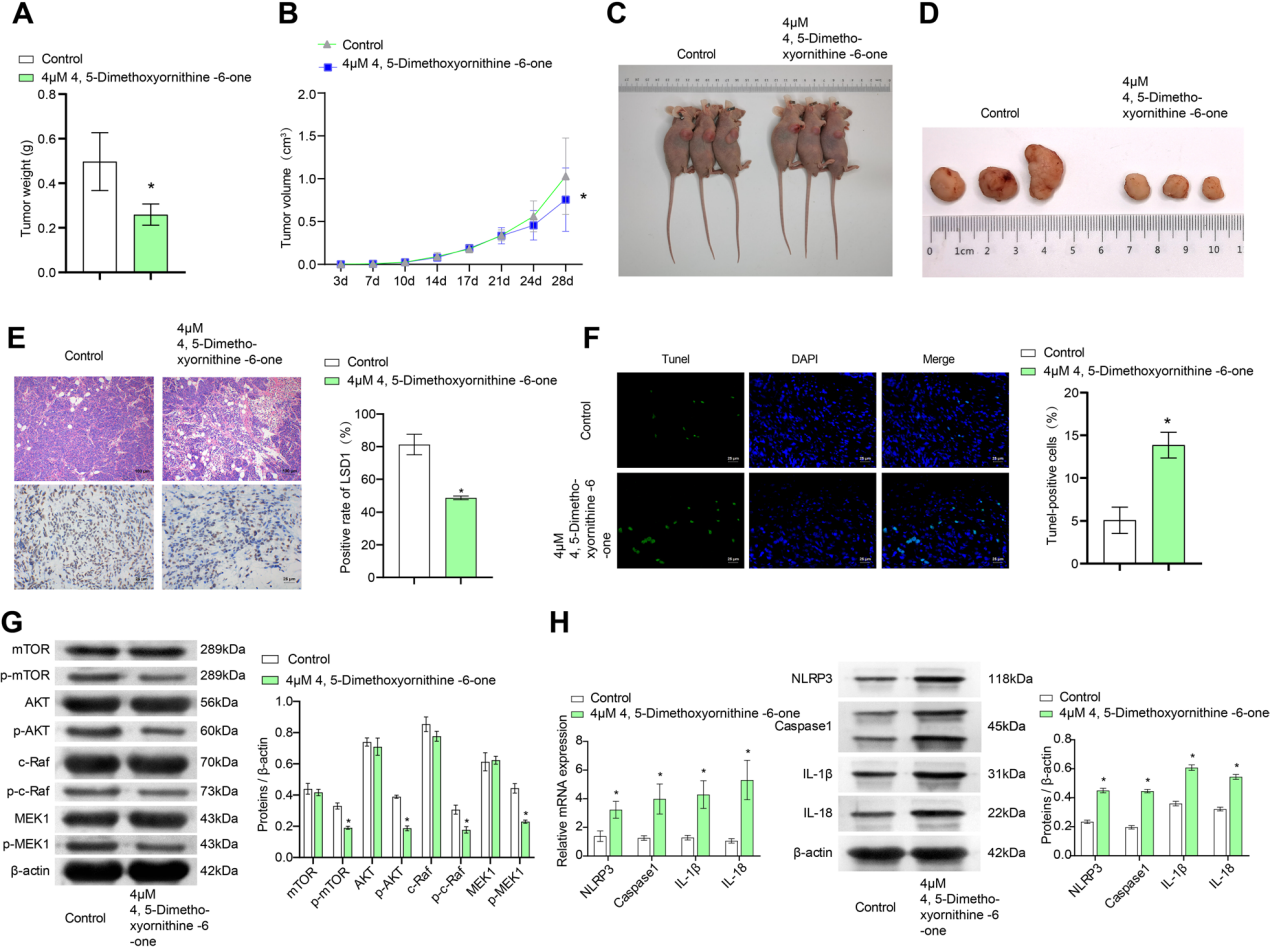
In vivo anti-tumor effects of 4, 5-dimethoxycanthin-6-one. Tumor weight (**A**) and volume (**B**). **C**, **D** Images of animals (**C**) and tumors (**D**). **E** Up: HE staining, down: LSD1 positivity was detected by IHC. **F** Cell apoptosis was tested by the TUNEL assay. **G** 4, 5-Dimethoxycanthin-6-one could inhibit the AKT/mTOR and MAPK expression. **H** Expression levels of NLRP3, Caspase1, IL-1β, and IL-18 were measured by qRT-PCR and western blot. *P < 0.05 compared with the Control group

The foregoing results indicated that 4 μM 4, 5-Dimethoxycanthin-6-one had the best effect. Thus, this concentration was used to explore the effect of 4, 5-dimethoxycanthin-6-one on the growth of glioblastoma cells in vivo. U251 cells were injected into nude mice as the Control group. The treated mice received 4, 5-Dimethoxycanthin-6-one group following the subaxillary injection of U251 cells. Compared with the Control group, the tumor volume in the 4 μM 4, 5-dimethoxycanthin-6-one group and the tumor mass were significantly reduced (Fig. 7A–D). Hematoxylin and eosin staining of tumor tissue in each group revealed that nuclei in the 4 μM 4, 5-dimethoxycanthin-6-one group was lighter in coloration, and cell arrangement was looser. IHC was used to detect the positive rate of LSD1 (Fig. 7E). The results showed that the positive rate of LSD1 was downregulated by 4 μM 4, 5-dimethoxycanthin-6-one (brown-yellow granules). The apoptosis index of the tumor tissue was determined by the TUNEL assay. Compared with the Control group, the number of TUNEL-positive cells increased in the 4 μM 4, 5-dimethoxycanthin-6-one group (Fig. 7F). qRT-PCR and western blot assays were used to detect cell pyroapoptosis and inflammatory signaling pathways, respectively. Expression of NLRP3, Caspase 1, IL-1β, and IL-18 increased in the 4 μM 4, 5-dimethoxycanthin-6-one group compared with that in the Control group. 4, 5-dimethoxycanthin-6-one had no obvious effect on the expression of mammalian target of mTOR, AKT, c-Raf, and MEK1. The level of p-mTOR, p-AKT,p-cRaf and p-MEK1 decreased in the 4 μM 4, 5-dimethoxycanthin-6-one group compared with that in the Control group (Fig. 7G, H). The collective results demonstrated that 4, 5-dimethoxyornithine-6-one significantly inhibited tumor growth.

## Discussion

The antitumor effect of 4, 5-dimethoxycanthin-6-one has been observed in a variety of tumors, including leukemia, colon cancer, retinoblastoma, and breast cancer [30]. 4, 5-Dimethoxycanthin-6-one is a natural medicinal extract that has low toxicity in animals. Its specificity to tumor tissue is different from that of chemically synthesized drugs [31]. Therefore, knowledge of the molecular understanding of the antitumor properties of 4, 5-dimethoxycanthin-6-one may be valuable for the treatment of glioblastoma. In the present study, 4, 5-dimethoxycanthin-6-one inhibited LSD1 methylation in T98G and U251 cells, which in turn inhibited the activation of the downstream AKT/mTOR and MAPK signaling pathways. The inhibition of these pathways triggered cell apoptosis and pyroptosis. This may be the main reason why 4, 5-dimethoxycanthin-6-one regulated the growth and metastasis of T98G and U251 cells. The collective findings indicate that the novel LSD1 inhibitor 4, 5-dimethoxycanthin-6-one slows down cell proliferation and migration, thus inhibiting the growth of glioblastoma cells in vitro and in vivo, and also induces pyroptosis.

Research on LSD1 function has focused on tumors [32]. The demonstration that LSD1 combines catalyzed the demethylation of H3K9 has increased research interest in the role of LSD1 in hormone-related tumors such as prostate cancer and breast cancer [33, 34]. Successive studies have demonstrated changes in LSD1 expression in nervous system tumors, bladder cancer, colon cancer, and many other types of tumors [35, 36]. Therefore, some LSD1 inhibitors have been investigated as potential antitumor drugs [37]. The expression of LSD1 increases in neuroblastoma tissues and cell lines [38, 39], and LSD1 is related to the degree of tumor malignancy [40]. The report have described a novel dual role for the histone demethylase LSD1 in controlling cell death and innate immune responses against pHGG and DIPG cells in vitro and in vivo [41]. The study findings elucidate a mechanism whereby LSD1 controls senescence in Glioblastoma tumor cells through the regulation of HIF-1α, and their propose the novel defined LSD1/HIF-1α axis as a new target for the therapy of Glioblastoma tumors [16]. In the present study, both in vivo and in vitro experiments confirmed that LSD1 could inhibit tumor growth after the expression of LSD1 was reduced or its effect was weakened. LSD1 is thus a key factor in maintaining the poorly differentiated malignant phenotype of neuroblastoma. Inhibition of LSD1 could reprogram cells to inhibit the growth of tumor cells, providing a new option for the treatment of neuroblastoma. We are not sure which mark is responsible to regulate LSD1 expression. We will study H3K4me1/2 and H3K9me2 in more depth in future studies.

Pyroptosis has become an increasingly common form of programmed cell death that occurs in biological scenarios, including tumor treatment and chronic inflammation [42]. Many studies have shown that LSD1 is highly expressed in normal tissues. However, LSD1 is activated by hypermethylation of the promoter in cancer cells [43]. This expression pattern is consistent with its role as a putative tumor suppressor. The reduction in LSD1 expression may represent a unique opportunity to utilize pyrophosphorylation to treat glioblastoma. In the present study, when LSD1 expression was blocked in vitro, the pyroptosis of glioblastoma cells increased. In vivo, combination therapy using a mouse xenograft model significantly improved survival. The released cell contents have the potential to stimulate inflammation and may initiate an antitumor immune response. We explored the role of glioblastoma apoptosis and pyroptosis, two mechanisms that could lead to programmed cell death. 4, 5-Dimethoxycanthin-6-one could reduce cell proliferation and cell cycle arrest, and increased cell apoptosis by targeting the LSD1 mediated AKT/mTOR and MAPK signaling pathways in glioblastoma. In some cell lines, pyroptosis has been shown to be a necrosis that is secondary to apoptosis. Pyroptosis is a mode of inflammatory cell death. IL-1β and IL-18 are pro-inflammatory cytokines that cause complex immune diseases. Compared with other cytokines, the secretion of IL-1β and IL-18 requires a second stimulus, which activates the inflammasome Caspase1 pathway and cleaves pro-IL-1β and IL-18 into mature and secretable forms [44]. Therefore, pyroptosis could have a synergistic effect with current antitumor immunotherapy. In addition, some tumor cells have anti-pyroptosis mechanisms, and the pyroptosis pathway may be an important way for drugs to kill tumor cells. Our results demonstrate that the expression of NLRP3, Caspase 1, IL-1β, and IL-18 was increased in the 4 μM 4, 5-dimethoxycanthin-6-one group compared with that in the Control group. 4, 5-dimethoxyornithine-6-one significantly inhibited tumor growth. Our limitation is that we haven't done clinical studies. We plan more in-depth explorations of the mechanism of pyroptosis. In the follow-up, we will further examine the effect of 4, 5-dimethoxyornithine-6-one on pyroptosis, and strive for clinical progress.

In conclusion, 4, 5-dimethoxycanthin-6-one inhibits the proliferation of glioblastoma cells in vitro and inhibits tumor growth in vivo. 4, 5-Dimethoxycanthin-6-one also promotes apoptosis of glioblastoma cells. High levels of 4, 5-dimethoxycanthin-6-one can induce Caspase 1 expression, which eventually triggers the pyrogen death of glioblastoma cells.

## Supplementary Information

**Additional file 1:** **Fig. S1**: Hypothesis figure.

**Additional file 2:** **Fig. S2**. The activity of HKF cell lines in different dose of 4, 5-dimethoxycanthin-6-One.

**Additional file 3: Fig. S3**. The UCSC genome browser and the chromatin enrichment marks on LSD promoter.

**Additional file 4: Fig. S4**: Annexin V-FITC and PI detection of the rate of cell apoptosis.

**Additional file 5:** **Table S1.** LSD1 siRNA sequences.

## Data Availability

All data in this article are true and reliable. You can contact the corresponding author for release.

## References

[1] Shergalis A, Bankhead A. Current challenges and opportunities in treating glioblastoma. Pharmacol Rev. 2018;70(3):412–45. 29669750 10.1124/pr.117.014944 PMC5907910

[2] Liu J, Jiang J. Mir-758-5p suppresses glioblastoma proliferation, migration and invasion by targeting ZBTB20. Cell Physiol Biochem. 2018;48(5):2074–83. 30099442 10.1159/000492545

[3] Abels ER, Maas SLN. Glioblastoma-associated microglia reprogramming is mediated by functional transfer of extracellular miR-21. Cell Rep. 2019;28(12):3105–19. 31533034 10.1016/j.celrep.2019.08.036 PMC6817978

[4] Liu Q, Guan Y. *miR-504 *suppresses mesenchymal phenotype of glioblastoma by directly targeting the FZD7-mediated Wnt–β-catenin pathway. J Exp Clin Cancer Res. 2019;2019(38). 10.1186/s13046-019-1370-1 PMC6697940 31419987

[5] Eckert M, Klumpp L. Cellular effects of the antiepileptic drug valproic acid in glioblastoma. Cell Physiol Biochem. 2017;44:1591–605. 29212069 10.1159/000485753

[6] Rhun EL, Preusser M. Molecular targeted therapy of glioblastoma. Cancer Treat Rev. 2019;80:101896. 31541850 10.1016/j.ctrv.2019.101896

[7] Engel M, Gee YS. Novel dual-action prodrug triggers apoptosis in glioblastoma cells by releasing a glutathione quencher and lysine-specific histone demethylase 1A inhibitor. J Neurochem. 2019;149(4):535–50. 30592774 10.1111/jnc.14655 PMC6590141

[8] Kurup JT, Kidder BL. Identification of H4K20me3- and H3K4me3-associated RNAs using CARIP-Seq expands the transcriptional and epigenetic networks of embryonic stem cells. J Biol Chem. 2018;293(39):15120–35. 30115682 10.1074/jbc.RA118.004974 PMC6166716

[9] Yamamoto J, et al. Histone methylation status of H3K4me3 and H3K9me3 under methionine restriction is unstable in methionine-addicted cancer cells, but stable in normal cells. Biochem Biophys Res Commun. 2020;533(4):1034–8. 33019978 10.1016/j.bbrc.2020.09.108

[10] Benard A, et al. Histone trimethylation at H3K4, H3K9 and H4K20 correlates with patient survival and tumor recurrence in early-stage colon cancer. BMC Cancer. 2014;14:531. 25047223 10.1186/1471-2407-14-531 PMC4223547

[11] Maiques-Diaz A, Somervaille TC. LSD1: biologic roles and therapeutic targeting. Epigenomics. 2016;8(8):1103–16. 27479862 10.2217/epi-2016-0009 PMC5066116

[12] Hosseini A, Minucci S. A comprehensive review of lysine-specific demethylase 1 and its roles in cancer. Epigenomics. 2017;9(8):1123–42. 28699367 10.2217/epi-2017-0022

[13] Wang M, Liu X. Downregulation of lysine-specific demethylase 1 enhances the sensitivity of hormone-sensitive prostate cancer cells to androgen deprivation therapy. Oncol Lett. 2021;21(2):93. 33376526 10.3892/ol.2020.12354 PMC7751335

[14] Castex J, Willmann D. Inactivation of Lsd1 triggers senescence in trophoblast stem cells by induction of Sirt4. Cell Death Dis. 2017;8(2):e2631. 28230862 10.1038/cddis.2017.48 PMC5386490

[15] Duteil D, Tosic M. Lsd1 prevents age-programed loss of beige adipocytes. Proc Natl Acad Sci USA. 2017;114(20):5265–70. 28461471 10.1073/pnas.1702641114 PMC5441764

[16] Saccà CD, et al. Inhibition of lysine-specific demethylase LSD1 induces senescence in Glioblastoma cells through a HIF-1α-dependent pathway. Biochim Biophys Acta Gene Regul Mech. 2019;1862(5):535–46. 30951900 10.1016/j.bbagrm.2019.03.004

[17] Lin Y, Kang T, Zhou BP. Doxorubicin enhances Snail/LSD1-mediated PTEN suppression in a PARP1-dependent manner. Cell Cycle. 2014;13(11):1708–16. 24675890 10.4161/cc.28619 PMC4111717

[18] Feng S, et al. Lysine-specific demethylase 1 (LSD1) inhibitor S2101 induces autophagy via the AKT/mTOR pathway in SKOV3 ovarian cancer cells. Med Sci Monit. 2016;22:4742–8. 27914215 10.12659/MSM.898825 PMC5142589

[19] Hou G, et al. LSD1 regulates Notch and PI3K/Akt/mTOR pathways through binding the promoter regions of Notch target genes in esophageal squamous cell carcinoma. Onco Targets Ther. 2019;12:5215–25. 31308693 10.2147/OTT.S207238 PMC6613024

[20] Rosina E, et al. Disruption of mTOR and MAPK pathways correlates with severity in idiopathic autism. Transl Psychiatry. 2019;9(1):50. 30705255 10.1038/s41398-018-0335-z PMC6355879

[21] Hachim MY, Khalil BA. Pyroptosis: the missing puzzle among innate and adaptive immunity crosstalk. J Leukoc Biol. 2020;108(1):323–38. 32083338 10.1002/JLB.3MIR0120-625R

[22] Wang K, Sun Q. Structural mechanism for GSDMD targeting by autoprocessed caspases in pyroptosis. Cell. 2020;180(5):941–55. 32109412 10.1016/j.cell.2020.02.002

[23] Miao X, Wang J. Identification of in vivo and in vitro metabolites of 4,5-dimethoxycanthin-6-one by HPLC-Q-TOF-MS/MS. J Chromatogr B Analyt Technol Biomed Life Sci. 2016;1020:78–84. 27030894 10.1016/j.jchromb.2016.03.011

[24] Miao X, Wang J. Pharmacokinetics and tissue distribution of 4,5-dimethoxycanthin-6-one and its major metabolites in rats. J Pharm Biomed Anal. 2017;139:22–9. 28258983 10.1016/j.jpba.2017.01.015

[25] Miao X, You J. In vitro metabolism of 4, 5-dimethoxycanthin-6-one by human liver microsomes and its inhibition on human CYP1A2. Life Sci. 2017;190:46–51. 28962866 10.1016/j.lfs.2017.09.031

[26] Kozono D, Li J. Dynamic epigenetic regulation of glioblastoma tumorigenicity through LSD1 modulation of MYC expression. Proc Natl Acad Sci USA. 2015;112(30):E4055–64. 26159421 10.1073/pnas.1501967112 PMC4522819

[27] Luo W, Li X. Long non-coding RNA AGAP2-AS1 exerts oncogenic properties in glioblastoma by epigenetically silencing TFPI2 through. Aging (Albany NY). 2019;11(11):3811–23. 31186379 10.18632/aging.102018 PMC6594811

[28] Singh MM, Manton CA. Inhibition of LSD1 sensitizes glioblastoma cells to histone deacetylase inhibitors. Neuro Oncol. 2011;13(8):894–903. 21653597 10.1093/neuonc/nor049 PMC3145466

[29] Yi L, Cui Y. Stabilization of LSD1 by deubiquitinating enzyme USP7 promotes glioblastoma cell tumorigenesis and metastasis through suppression of the p53 signaling pathway. Oncol Rep. 2016;36(5):2935–45. 27632941 10.3892/or.2016.5099

[30] Shi Y, Hong C. Simultaneous quantification of two canthinone alkaloids of Picrasma quassioides in rat plasma by liquid chromatography–tandem mass spectrometry and its application to a rat pharmacokinetic study. J Chromatogr B Analyt Technol Biomed Life Sci. 2015;1:986–7. 10.1016/j.jchromb.2015.02.008 25725320

[31] Chen L, Miao X. The pharmacokinetics and bioavailability of three canthinone alkaloids after administration of Kumu injection to rats. J Ethnopharmacol. 2016;182:235–41. 26806576 10.1016/j.jep.2016.01.019

[32] Gu F. Biological roles of LSD1 beyond its demethylase activity. Cell Mol Life Sci. 2020;77(17):3341–50. 32193608 10.1007/s00018-020-03489-9 PMC11105033

[33] Gao S, Chen S. Chromatin binding of FOXA1 is promoted by LSD1-mediated demethylation in prostate cancer. Nat Genet. 2020;52(10):1011–7. 32868907 10.1038/s41588-020-0681-7 PMC7541538

[34] Cuyàs E, Gumuzio J. The LSD1 inhibitor iadademstat (ORY-1001) targets SOX2-driven breast cancer stem cells: a potential epigenetic therapy in luminal-B and HER2-positive breast cancer subtypes. Aging (Albany NY). 2020;12(6):4794–814. 32191225 10.18632/aging.102887 PMC7138538

[35] Xie Q, Tang T. LSD1 Promotes Bladder Cancer Progression by Upregulating LEF1 and Enhancing EMT. Front Oncol. 2020;10. 10.3389/fonc.2020.01234 PMC7399223 32850370

[36] Chen J, Ding J. Identification of downstream metastasis-associated target genes regulated by LSD1 in colon cancer cells. Oncotarget. 2020;8(12):19609–30. 10.18632/oncotarget.14778 PMC5386709 28121627

[37] Fang Y, Liao G. LSD1/KDM1A inhibitors in clinical trials: advances and prospects. J Hematol Oncol. 2019;12(1). 10.1186/s13045-019-0811-9 PMC6894138 31801559

[38] Lu Y, et al. LncRNA HAS2-AS1 Promotes Glioblastoma Proliferation by Sponging miR-137. Front Oncol. 2021;11:634893. 34094916 10.3389/fonc.2021.634893 PMC8173206

[39] Singh MM, et al. Inhibition of LSD1 sensitizes glioblastoma cells to histone deacetylase inhibitors. Neuro Oncol. 2011;13(8):894–903. 21653597 10.1093/neuonc/nor049 PMC3145466

[40] Zhao J, et al. Combination LSD1 and HOTAIR-EZH2 inhibition disrupts cell cycle processes and induces apoptosis in glioblastoma cells. Pharmacol Res. 2021;171:105764. 34246782 10.1016/j.phrs.2021.105764

[41] Bailey CP, et al. Pharmacologic inhibition of lysine-specific demethylase 1 as a therapeutic and immune-sensitization strategy in pediatric high-grade glioma. Neuro Oncol. 2020;22(9):1302–14. 32166329 10.1093/neuonc/noaa058 PMC7523459

[42] Shi J, Gao W. Pyroptosis: gasdermin-mediated programmed necrotic cell death. Trends Biochem Sci. 2017;42(4):245–54. 27932073 10.1016/j.tibs.2016.10.004

[43] Zhang H, Gao Q. SET8 prevents excessive DNA methylation by methylation-mediated degradation of UHRF1 and DNMT1. Nucleic Acids Res. 2019;47(17):9053–68. 31400111 10.1093/nar/gkz626 PMC6753495

[44] Wang J, Sahoo M. Caspase-11-dependent pyroptosis of lung epithelial cells protects from melioidosis while caspase-1 mediates macrophage pyroptosis and production of IL-18. PLoS Pathog. 2018;14(5):e1007105. 29791511 10.1371/journal.ppat.1007105 PMC5988316

