# Correction: 4,5-Dimethoxycanthin-6-one is a novel LSD1 inhibitor that inhibits proliferation of glioblastoma cells and induces apoptosis and pyroptosis

**DOI:** 10.1186/s12935-024-03311-7

**Published:** 2024-09-14

**Authors:** Wei Li, Bai-Sheng Huang, Yuan-Yuan Xiong, Li-Jian Yang, Li-Xiang Wu

**Affiliations:** 1 Department of Physiology, School of Basic Medical Sciences, Central South University, 110 Xiangya Road, Changsha City, Hunan Province China; 2 Department of Neurosurgery, The Second Affiliated Hospital of Nanchang University, Nanchang, China; 3 Department of Neurosurgery, The First Hospital of Changsha, Changsha, China

**Correction to: Cancer Cell International (2022) 22:32** 10.1186/s12935-021-02434-5

In this article [1], the WB band for mTOR in Fig. 3B and the Control group of T98G cells in Fig. 4A were incorrect. The corrected Figs. 3 and 4 are given below.

**Fig. 3:**
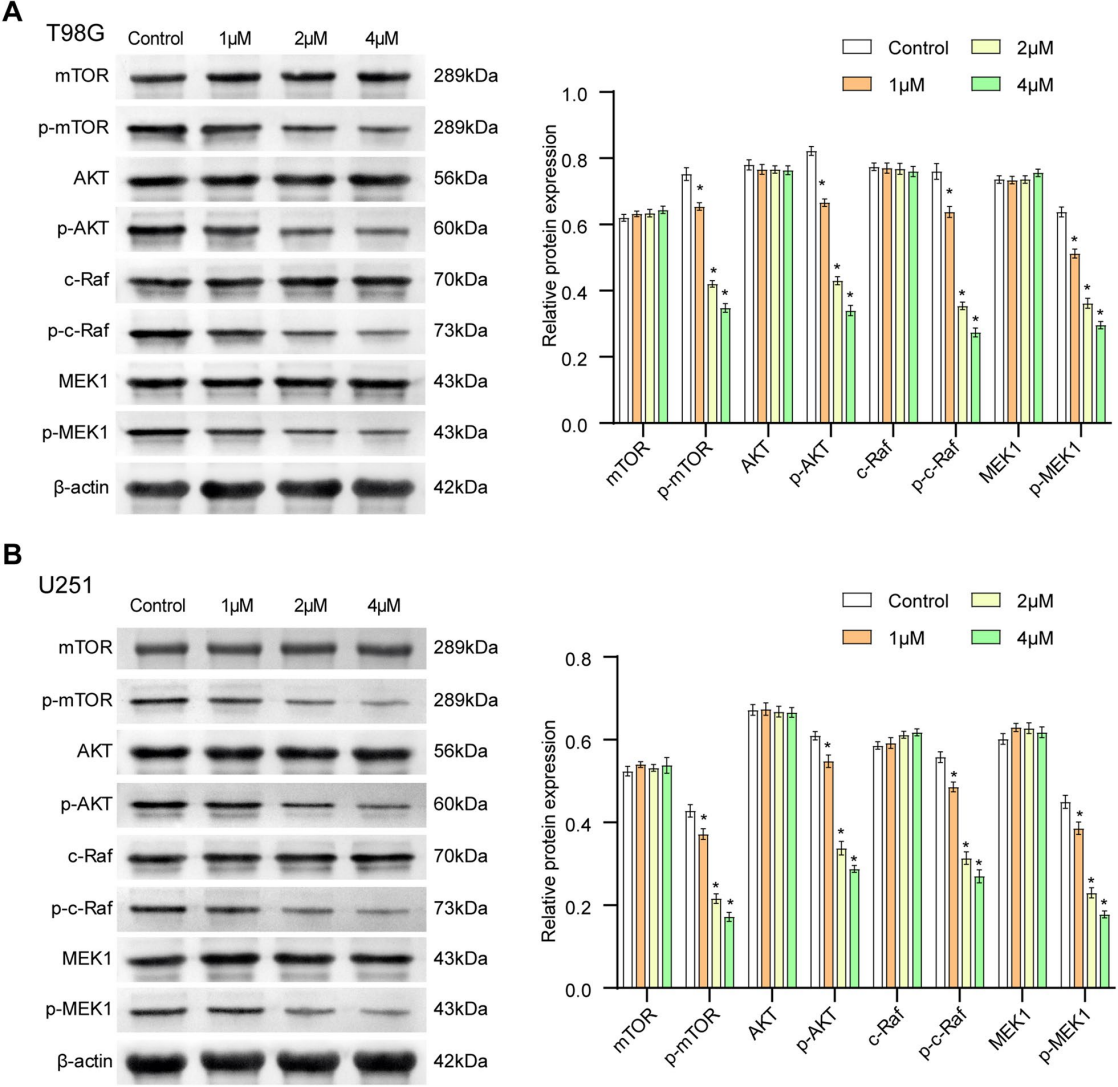
4, 5-Dimethoxycanthin-6-one inhibits the AKT/mTOR and MAPK signaling pathways. **A** 4, 5-Dimethoxycanthin-6-one inhibition of the AKT/mTOR and MAPK signaling pathways in U251 cells. **B** 4, 5-Dimethoxycanthin-6-one inhibition of the AKT/mTOR and MAPK signaling pathways in T98G cells. *P < 0.05 compared with the Control group

**Fig. 4:**
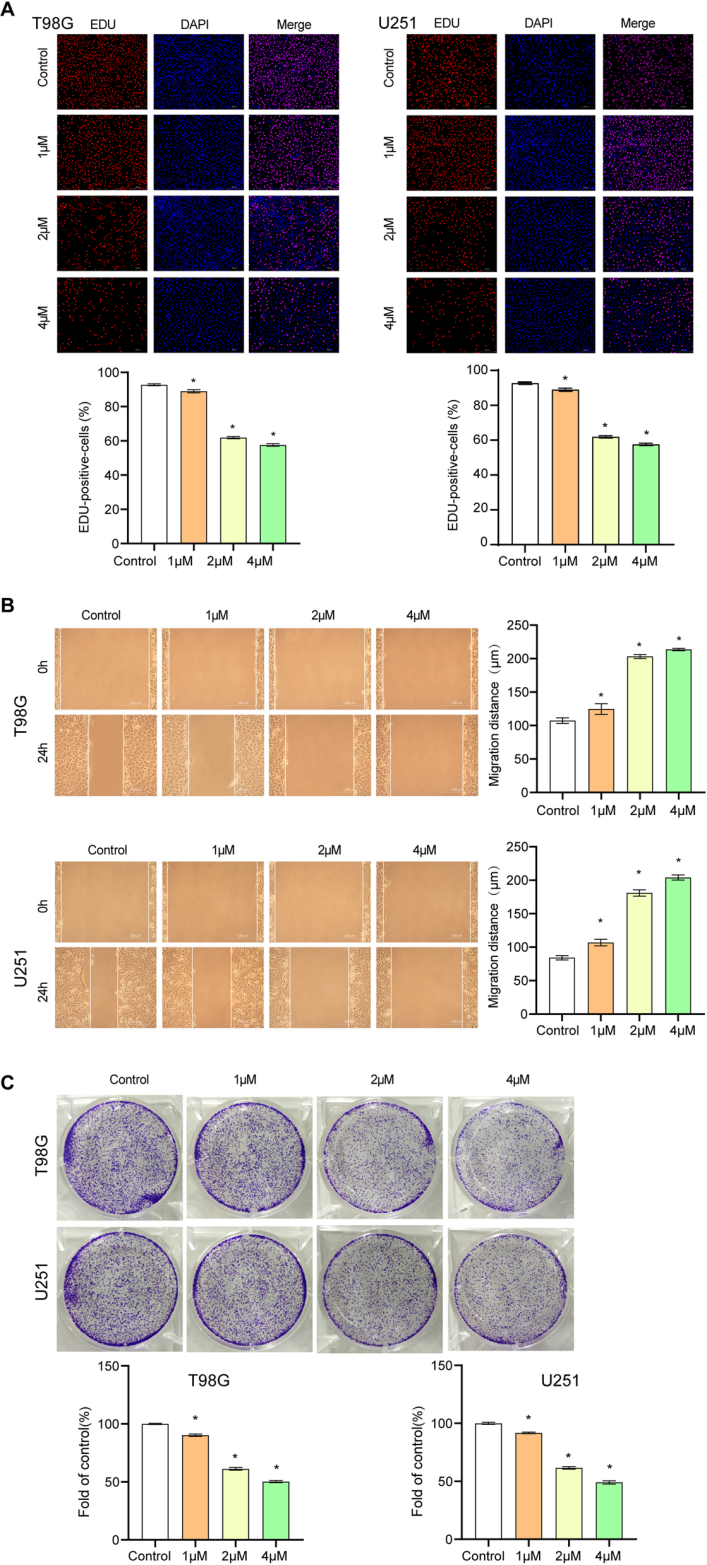
4, 5-Dimethoxycanthin-6-one inhibits cell proliferation. **A** Cell proliferation detected using the EDU assay. **B** The migration distance of cells was measured using a wound scratch assay. **C** Colon numbers were analyzed using a colony formation assay. *P < 0.05 compared with the Control group
