# Supplementary figures and images for: 4,5-Dimethoxycanthin-6-one is a novel LSD1 inhibitor that inhibits proliferation of glioblastoma cells and induces apoptosis and pyroptosis
Source: Cancer Cell Int. 2022 Jan 18;22:32. doi: 10.1186/s12935-021-02434-5 (PMC8764814; doi:10.1186/s12935-021-02434-5)

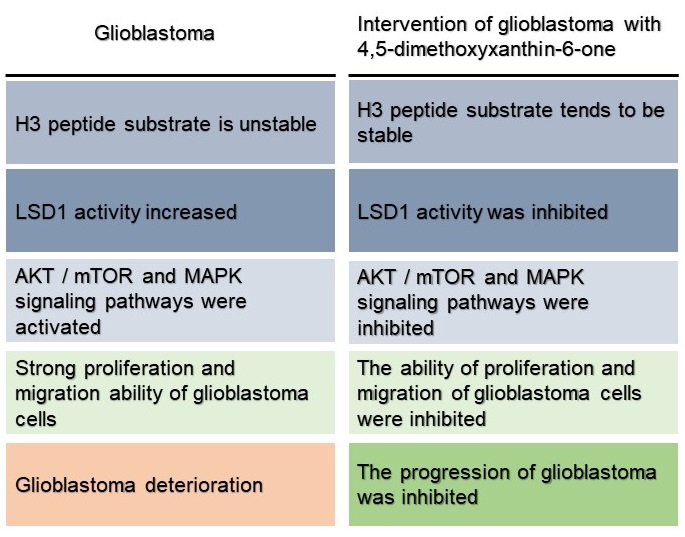

Supplement: Supplementary file 1 — Additional file 1: Fig. S1: Hypothesis figure. [file 12935_2021_2434_MOESM1_ESM.jpg]

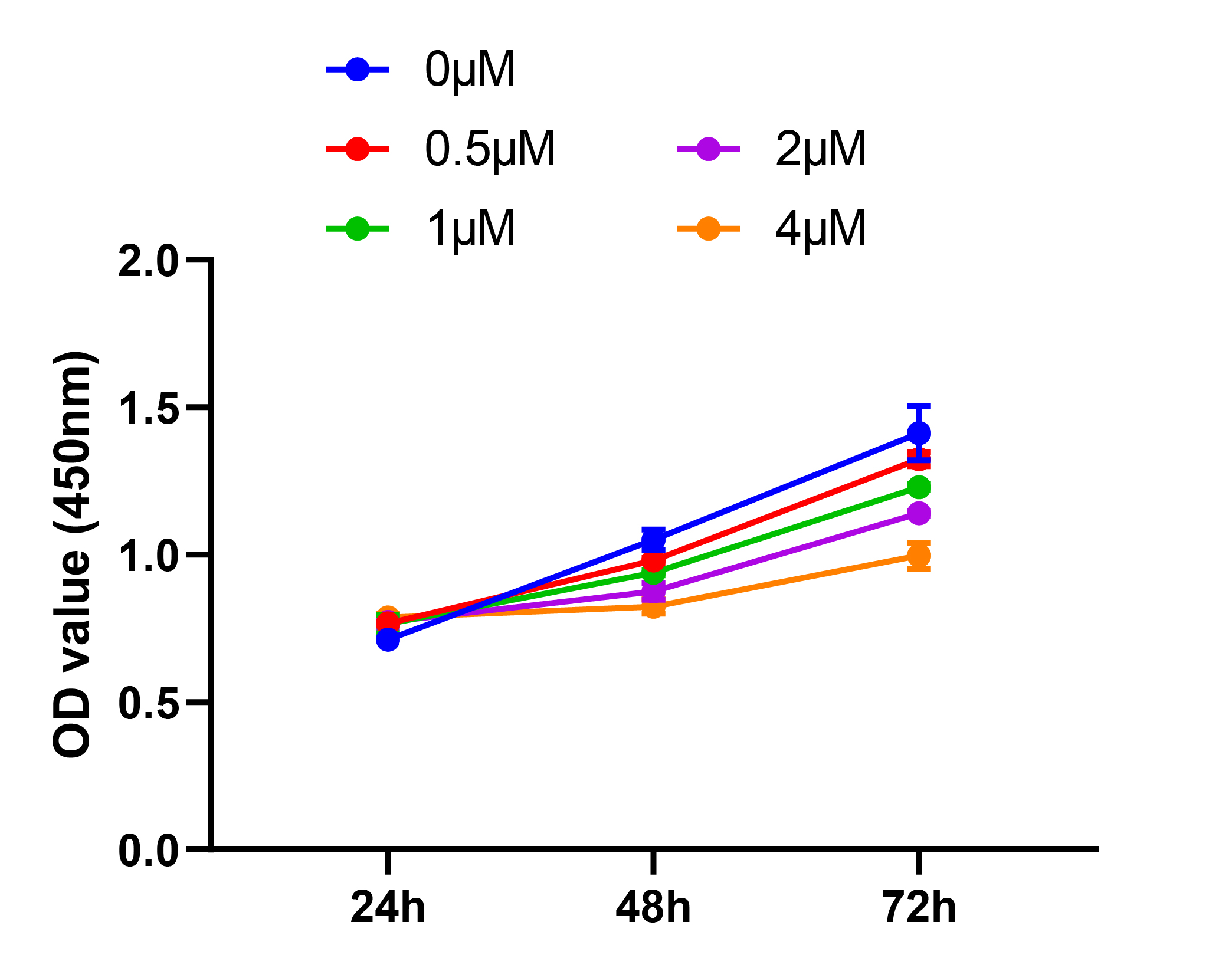

Supplement: Supplementary file 2 — Additional file 2: Fig. S2. The activity of HKF cell lines in different dose of 4, 5-dimethoxycanthin-6-One. [file 12935_2021_2434_MOESM2_ESM.jpg]

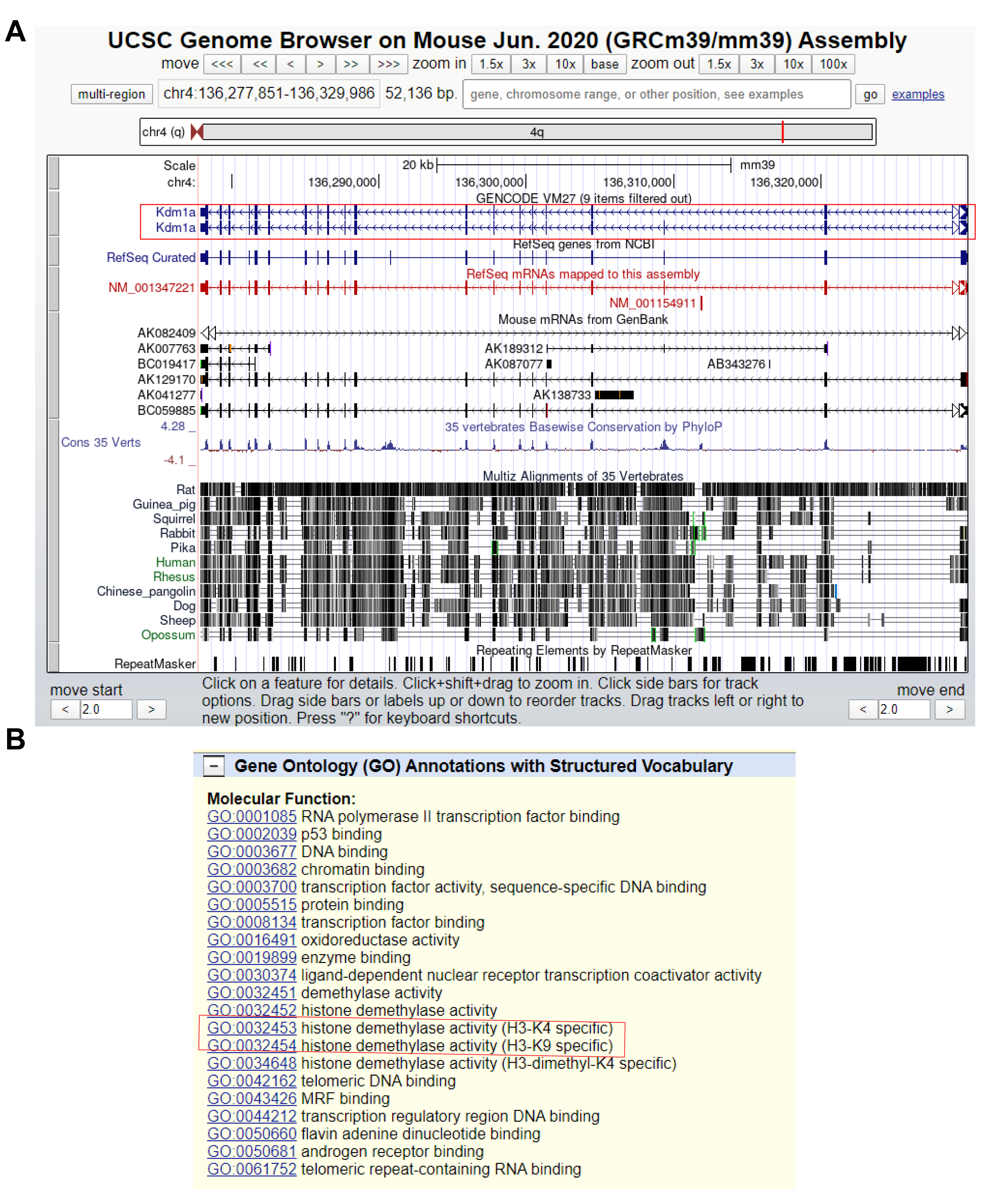

Supplement: Supplementary file 3 — Additional file 3: Fig. S3. The UCSC genome browser and the chromatin enrichment marks on LSD promoter. [file 12935_2021_2434_MOESM3_ESM.jpg]

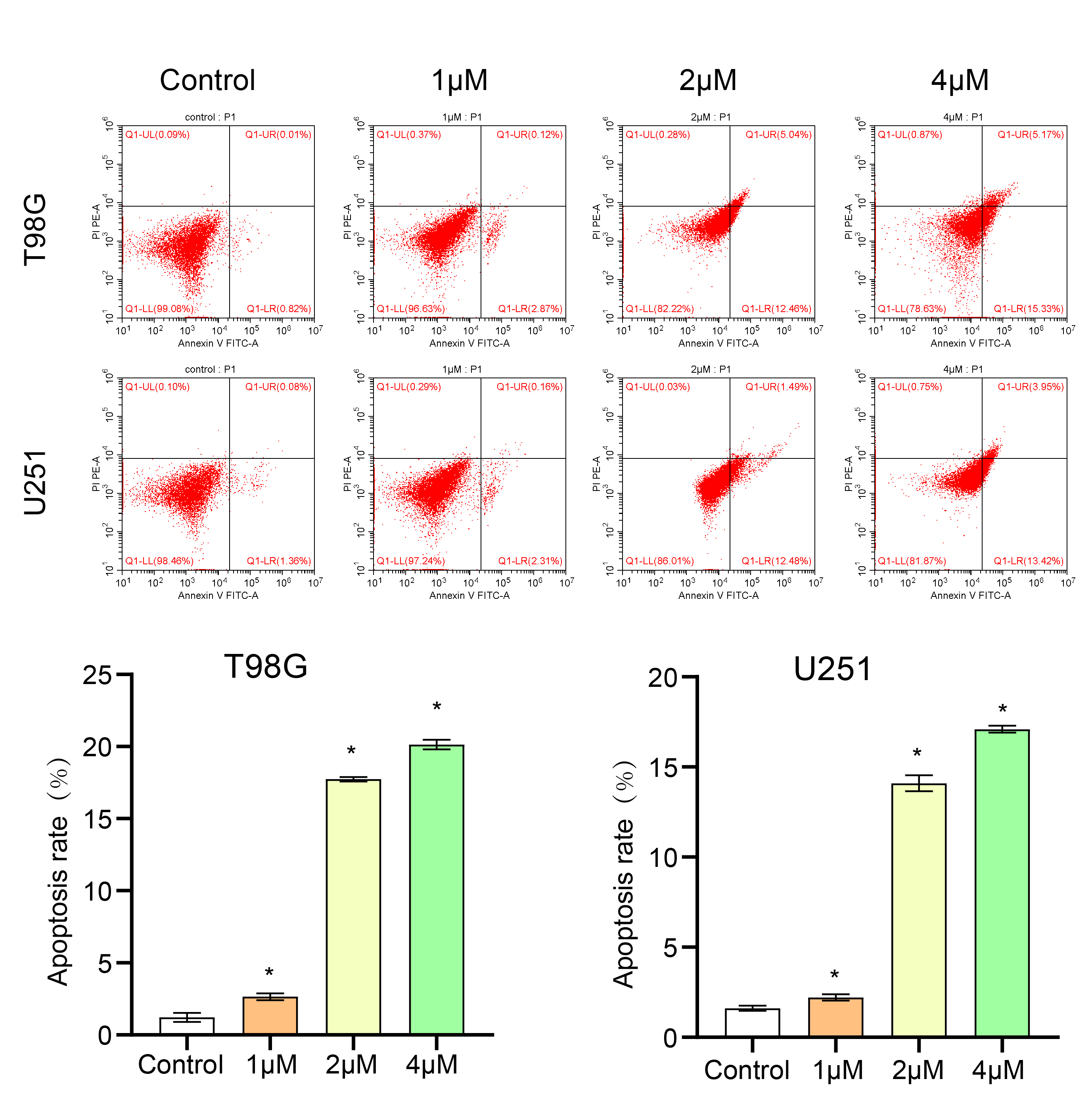

Supplement: Supplementary file 4 — Additional file 4: Fig. S4: Annexin V-FITC and PI detection of the rate of cell apoptosis. [file 12935_2021_2434_MOESM4_ESM.jpg]
