# Supplementary material for: 4,5-Dimethoxycanthin-6-one is a novel LSD1 inhibitor that inhibits proliferation of glioblastoma cells and induces apoptosis and pyroptosis
Source: Cancer Cell Int. 2022 Jan 18;22:32. doi: 10.1186/s12935-021-02434-5 (PMC8764814; doi:10.1186/s12935-021-02434-5)
Supplement: Supplementary file 5 — Additional file 5: Table S1. LSD1 siRNA sequences. [file 12935_2021_2434_MOESM5_ESM.docx]

**Table S1: LSD1 siRNA sequences**

| sites | Sequences |
| --- | --- |
| Target sequence 1 | CUAUAAAGCUCCAAUACUG |
| Target sequence 2 | GUAAAGCCACCCAGAGAUA |
| Target sequence 3 | GACAAGCUGUUCCUAAAGA |
